# Supplementary material for: Are carbon emissions trading and green financial instruments synergistic? -Comprehensive quantitative research based on content analysis
Source: PLoS One. 2024 Mar 7;19(3):e0298601. doi: 10.1371/journal.pone.0298601 (PMC10919601; doi:10.1371/journal.pone.0298601)
Supplement: S1 Data — (ZIP) [file pone.0298601.s001.zip › Data/地级市Z_F工作报告.zip!/║■▒▒/╗╞╕╘╩╨/└┤╫╘╣1⁄2╓┌║┼í╛┬φ┐╦╩2╛▌═°í┐_2010.docx]

各位代表：

　　现在，我代表市人民政府向大会报告工作，请予审议，并请市政协各位委员和其他列席人员提出意见。

　　一、2009年工作回顾

　　2009年是新世纪以来黄冈经济发展最困难的一年，也是我市历史上发展最好的年份之一。在省委、省政府和市委的坚强领导下，市政府团结带领全市人民，坚定信心，迎难而上，全力保增长、保民生、保稳定，各项工作取得显著成绩，较好地完成了市三届人大四次会议确定的目标任务。

　　——经济平稳较快增长。全市地区生产总值700.3亿元，增长15%;规模以上工业增加值192.9亿元，增长27.6%;全口径财政收入75亿元，增长25.9%;地方一般预算收入32.2亿元，增长22.7%。除外贸出口外，经济社会发展预期目标全面超额完成。主要经济指标增幅继续保持全省领先。规模以上工业企业户数及规模以上工业增加值、全社会固定资产投资及城镇以上固定资产投资、全口径财政收入及地方一般预算收入，均在2006年的基础上实现了翻番。财政收入、固定资产投资等10项指标提前两年，地区生产总值和社会消费品零售总额提前一年完成“十一五”规划预期目标。

　　——可持续发展能力增强。全社会固定资产投资553.3亿元，增长49.2%，城镇以上固定资产投资484.6亿元，增长53.4%。单位生产总值能耗下降6.74%，化学需氧量和二氧化硫排放量分别下降2.51%和0.83%。

　　——民生质量提高。新增城镇就业5.1万人，城镇登记失业率为4.25%。城镇居民人均可支配收入11336元，增长13.9%;农民人均纯收入4130元，增长9.4%。

　　一年来，我们主要做了以下工作：

　　(一)全力以赴保增长

　　扎实开展“大干项目年”活动。全市在建项目3273个，同比增长51%，其中新开工项目2913个，投产项目2099个;亿元以上在建项目112个，列入国家、省级重点项目14个。获得中央新增投资项目1309个、资金15.2亿元。大广、武英高速公路建成通车。长江回水堤防整险加固工程全面竣工。武汉至黄冈城际铁路、黄冈长江大桥和黄鄂高速公路动工兴建。大别山旅游公路、黄州火车站扩建改造、武麻高速、麻竹高速、九江长江二桥北岸接线、祥云和广药产业园、鸿路钢构、东盟产业园、李时珍国际医药港等重点项目顺利推进。黄冈电厂、林纸一体化、浠水核电等项目前期工作取得重要进展。

　　千方百计保企业。实施支持中小企业稳定健康发展的优惠政策措施，组织开展“大调研大服务”活动和中小企业银企合作促进行动，尽心竭力帮助企业渡过难关。为企业落实政策性减税4.2亿元，新增中小企业贷款27.1亿元，市县两级担保机构为200余家中小企业融资担保18.1亿元。支持企业上市，2家企业进入上市辅导期，9家企业正式启动或即将启动上市程序。

　　统筹区域经济发展。坚持高昂市区龙头，市直投入财政资金8776万元，全面启动南湖工业园和黄冈化工园建设;落实财税奖励资金2144万元，重点支持饮品、造船、窑炉、机械电子等产业发展;多方筹措资金1亿多元，扶持奶牛、蔬菜、水产等高效农业。完善考评指标体系，健全激励竞争机制，促进县域经济竞相发展。9个县市区工业增加值增幅达到20%以上，5个县市工业增加值突破20亿元。黄州、武穴进入全省县域经济20强、蕲春获最佳进位奖。

　　积极扩大消费需求。实施“万村千乡市场工程”，开展“家电、汽车下乡”和繁荣城乡市场系列活动，全市社会消费品零售总额340亿元，增长20.6%。旅游综合收入30亿元，增长20%。房地产投资增长36.4%，住房按揭贷款增长71%。实施出口多元化战略，有效应对外需紧缩，逐步收窄出口降幅。外贸出口4.06亿美元，同比下降18%。

　　全力保障要素供应。积极搭建信贷、融资、担保平台，全面推进政银企合作，金融机构各项贷款余额达到317.2亿元，当年新增64.8亿元，同比多增30.5亿元。市城投公司通过重组资产达到67亿元。加强煤电油气运的调度和管理，妥善解决发展用地制约。城乡电网建设投资6.6亿元，新设供电线路310公里，新增主变容量69.3万千伏安;工业用电量增长8.2%，高于全省4.8个百分点。

　　(二)着力推进产业优化升级

　　突出重点抓工业。大力推进园区建设，建成各类开发区和工业园区30个，规划面积257平方公里，建成区面积91平方公里，入园规模以上企业900多家。建成乡镇工业集中区75个，入区企业1969家。强化优势产业聚集效应，初步形成产业集群31个，其中7个被列为全省重点。造船业17家企业投产，产值增长2.1倍。钢构产业产值增长134.6%。节能灯、陶瓷、窑炉、森工等新兴产业迅猛发展。大力培育市场主体，规模以上工业企业发展到1348家，净增345家。产值过亿元企业发展到104家，新增34家。伊利乳业、开阳星造船等企业产值成倍增长。

　　加快发展现代服务业。制定出台一系列政策措施，着力推进总部经济、现代物流、商贸流通业快速发展。抢抓省政府大别山旅游开发现场办公会的机遇，组建黄冈大别山旅游开发公司，大力推进旅游资源整合。全市4A级景区发展到5家，3A级景区达到9家，红安七里坪镇跻身全省首批旅游名镇，罗田圣人堂等13个村被列为全省百家旅游名村。

　　加大科技创新和品牌建设力度。实施技改项目68个，投入技改资金43.8亿元，增长25%。拥有国家级高新技术企业11家、省级创新型试点企业7家。高新技术产业实现产值84.8亿元，比上年增长16.7%。再次荣获全国科技进步先进城市称号。实施质量兴市战略，新创建湖北名牌14个，新认证无公害农产品54个、国家地理标志保护产品9个。

　　毫不松懈地抓好“三农”工作。全市规模以上农产品加工企业发展到464家，比上年净增27家。建成省级以上重点龙头加工企业32家。发展农民专业合作社710家。特色农业板块基地面积达到700多万亩。建设高产农田24万亩，改造低丘岗地和低效林17.4万亩。粮棉油、畜禽、水产等大宗农产品和茶叶、板栗等特色农产品全面增产，奶牛存栏1.7万头。新建通村水泥路3791公里。投资18亿元，开工建设水利工程1.8万处，完成20座病险水库除险加固、9处大型排涝泵站更新改造，新解决农村66万人饮水安全问题。完善农业技术推广服务体系，提高农业机械化水平，发放农机补贴6340万元。移民工作取得新进展。

　　(三)积极推进改革试验和对外开放

　　全面启动临港经济区和沿江经济带建设。加快黄冈高新区、黄冈化工园、南湖工业园、禹王产业园等园区建设步伐。完成了沿江经济带规划论证和项目策划工作。武汉新港黄冈港区疏港通道建设加快推进，唐家渡综合码头建设基本完工。成功申报和启动建设大别山(黄冈)国家地质公园、龙感湖国家级湿地保护区和蕲春赤东湖国家级湿地公园。首次引资自主建设麻竹高速公路，首次采用BOT模式兴建黄鄂高速公路，首次采用BT模式启动市博物馆等五大社会事业项目建设。

　　强力推进节能减排。建立完善节能减排联席会议制等“五制联动”工作机制，创新实施“河湖库长”制，大力开展环保专项治理，圆满完成省政府下达的节能减排任务。市、县污水处理厂建设完成投资9.3亿元，3家投产运行，5家进入试运行。依法关闭“九小”企业104户。加强对冶金、建材、化工等重点行业的节能改造。蕲春县动建全省首个现代林业低碳经济产业园，龙感湖获批全省首家循环经济工业园。

　　稳步实施重点领域改革。全面启动政府机构改革，深入推进市县乡三级行政服务网络建设。加强国有资产经营监管，加快市属国有企业改制步伐，争取省级专项补助资金5000万元。深化集体林权制度改革，加快推进土地流转和规模经营，抓好城中村、城郊村、园中村集体资产产权制度创新工作。鼓励支持非公有制经济发展，当年新增个体工商户3.1万户、私营企业2009家。非公工业实现增加值147.5亿元，增长35.3%。

　　扩大对外开放。新引进项目1106个，投资规模748亿元，其中10亿元以上项目11个，招商引资新建、续建项目完成投资170.2亿元。实际利用外资1.5亿美元，增长15.1%。深入推进市厅、市校合作，与省直17个单位建立共建关系。

　　(四)努力提高城乡规划建设和管理水平

　　科学调整城乡建设规划。编制完成了市区及工业园区控制性详细规划、全市土地利用总体规划，整体推进县市城区、乡镇和村庄规划修编工作，在调整空间布局、推动集约发展方面取得突破性进展。与国家和省测绘局共建黄冈市区地理信息系统，“数字黄冈”建设迈出实质性步伐。

　　加强城乡基础设施建设。市区多方筹措资金4.8亿元，重点推进遗爱湖保护治理工程，大力开展清淤截污，高质量、高品位建设一批景点设施。完成道路刷黑12.7万平方米，道路硬化1.6万平方米，人行道铺装6.7万平方米，新增公共绿地42万平方米。以“楚天杯”创建为载体，加强县市城区建设;以实施“百镇千村”工程为抓手，有序推动小城镇建设和新农村建设，崛起了一批新型集镇。

　　提高城市管理水平。持续深入开展市区“四城联创”活动，着力整治市容市貌，切实规范城市交通秩序、市场秩序、卫生秩序和社会治安秩序，不断提升城市形象。成功创建省级卫生城市。

　　(五)切实加强以民生为重点的社会建设

　　扎实推进民生工程。实施“创业扶持工程”，落实“五缓四减三补贴”援企稳岗政策和小额担保贷款、职业技能培训等创业就业扶持政策，尽力解决返乡农民工和城镇人员的就业问题。认真办好团风县新农保试点。积极开展生育保险和在校学生参加城镇医疗保险，启动农业“小三场”职工、被征地农民、村主职干部、被辞退民办教师参加养老保险，社会保障水平有了新提高。完善社会救助体系，农村“五保”老人和散居孤儿供养新标准全面落实。加强保障性住房建设，完成廉租住房建设项目15个、29.1万平方米，建设规模和水平居全省前列。认真执行国家粮油最低收购价政策，力保农民增产增收。大力推进扶贫开发，448个重点村扶贫开发工作全面启动，5014户农村危房改造工作全面展开，21.66万贫困人口实现稳定脱贫。市政府承诺的10件为民办实事项目全面完成。

　　积极发展社会事业。实施义务教育均衡发展行动计划和义务教育教师绩效工资制度，有序推进中等职业教育资源整合，支持高等院校加快发展，教学质量稳步提高。深化医药卫生体制改革，全面落实城乡居民基本医疗保险制度，继续推进新型农村合作医疗制度建设，完善医疗救助办法，加强甲型H1N1流感等疫病防控工作。文化艺术活动丰富多彩，电影《黎明行动》荣获国家“五个一工程”奖，第七届黄梅戏艺术节成功举办。明、清《黄州府志》重刊出版。大力发展体育事业，荣获全国农民健身工程建设先进市和群众体育先进市称号。

　　强化社会管理。严格执行计划生育责任制和“一票否决制”，加大违反计划生育政策案件查处力度，人口和计生工作完成预定目标。深入推进“法治黄冈”、“平安黄冈”建设，加强社会治安综合治理，加大信访维稳工作力度，严厉打击各类违法犯罪，实现了当年命案全破的重大突破。基层应急体系建设扎实推进，电子政务专网投入运行。加强物价管理，居民消费价格指数下降0.5个百分点。食品药品和农产品安全监管成效明显。全面开展安全生产“三项行动”，加强重点领域安全整治和监管，加大消防工作力度，安全生产工作连续7年获得省政府表彰。圆满完成了汉源县地震灾区援建任务。

　　国防动员、“双拥”、民兵预备役、人民防空工作扎实推进。驻黄解放军、武警部队在地方发展中发挥了重要作用。

　　(六)努力建设服务型政府

　　突出“大干项目促三保，科学发展兴黄冈”实践主题，深入开展学习实践科学发展观活动，着力整改落实15件事关群众切身利益的问题。坚持依法行政，自觉接受人大的法律监督、工作监督和政协的民主监督，办理人大代表建议121件，政协委员提案258件，办复率均为100%。治理车辆超限超载、加强农村建房和用地管理两项议案办理工作取得阶段性成果。加强行政监察和审计监督，加强源头治腐和勤政廉政建设。统计、调查、气象、防震、民族、宗教、档案、保密、咨询、对台、外事、侨务等工作得到加强，妇女、儿童、老龄、残疾人事业健康发展。

　　各位代表，回顾过去的一年，成绩来之不易。这些成绩凝结着全市人民的心血和汗水，体现着社会各界的帮助和支持。在此，我代表市人民政府，向推动黄冈发展的全市人民，向大力支持政府工作的人大代表和政协委员，向各民主党派、工商联、人民团体和无党派人士，向离退休老同志，向驻黄部队和武警官兵，向来黄冈投资创业的中外客商，向关心支持黄冈建设发展的海内外朋友们，表示衷心的感谢!

　　在肯定成绩的同时，我们清醒地认识到，经济社会发展还面临不少困难和问题，政府工作还有不足之处：国际金融危机的不利影响仍在继续，我市经济增长基础尚不牢固，企业创新能力不足，外贸出口受阻，利用外资困难增多;“两型社会”建设任重道远，节能减排压力较大;农村基础设施建设滞后，农业产业化水平较低，农民持续增收难度加大;社会事业发展欠账较多，一些事关群众切身利益的问题比较突出;投资环境有待进一步优化，行政效能需要进一步提高。对此，我们将进一步增强忧患意识和责任意识，坚持应急与谋远相结合，远谋近施、长短兼顾，努力加以解决。

　　二、2010年政府工作的指导思想和奋斗目标

　　2010年是实施“十一五”规划的最后一年，是谋划“十二五”规划的奠基之年，也可能是我市经济发展最为复杂但又蕴含重要机遇的一年。虽然外部环境不确定不稳定因素仍然较多，但从总体上看，今年的经济发展环境将好于去年，黄冈仍处于重要战略机遇期。国家继续实施积极的财政政策和适度宽松的货币政策、省委省政府进一步加大武汉城市圈“两型社会”建设和新一轮长江经济带开放开发力度，为黄冈振兴发展注入了新的动力。我市多年积蓄的能量，为实现又好又快发展提供了强大的后劲。只要我们始终保持坚定的信念和昂扬向上的精神状态，把形势判断得更准确一些，把困难估计得更充分一些，把措施安排得更周密一些，高标准、高效率、高水平地抓好各项工作，就一定能够在新一轮增长中把握主动，就一定能够在后发赶超中大有作为，就一定能够在科学发展的实践中再创佳绩!

　　2010年政府工作的指导思想是：全面贯彻党的十七大、十七届三中、四中全会、中央和全省经济工作会议精神，深入实践科学发展观，把保持经济平稳较快增长作为首要任务，把实施“一区两带”战略作为关键举措，把结构调整和产业升级作为工作主线，把加强经济社会管理作为重要保障，把改善人民群众生活作为根本宗旨，加快推进工业化、城镇化、农业现代化，协调推动经济建设、政治建设、文化建设、社会建设和生态文明建设，确保完成和超额完成“十一五”各项目标任务，努力实现经济社会又好又快地发展。

　　2010年我市经济社会发展主要预期目标为：地区生产总值增长12%，规模以上工业增加值增长20%以上，全社会固定资产投资增长25%以上，社会消费品零售总额增长20%，实际利用外资增长8%，外贸出口增长10%，地方一般预算收入增长16%;单位生产总值能耗下降4%，化学需氧量和二氧化硫排放量分别下降2.5%和0.8%;城镇居民人均可支配收入增长10%，农民人均纯收入增长8%;城镇新增就业4万人，城镇登记失业率控制在4.5%以内，居民消费价格涨幅控制在3%左右;人口自然增长率控制在6‰以内。

　　三、加快结构调整和转变发展方式，着力提升经济发展质量和效益

　　深入开展大干项目提升年活动。努力扩大投资规模。健全项目策划、服务、考评、问责机制，切实提高项目核准率、落户率、资金到位率、开工竣工率和投产达产率。重点实施204个亿元项目尤其是18个国家级、省级重大项目，着力抓好武麻高速、麻竹高速、黄州火车站扩建改造、武汉新港黄冈港区、大别山旅游公路等续建项目。全力抓好武汉至黄冈城际铁路、黄冈长江大桥及黄鄂高速公路、九江长江二桥及北岸接线等工程建设的服务协调工作，高标准完成征地、拆迁和安置工作。强力推进黄冈电厂、大别山电厂二期、浠水核电、林纸一体化、长河流域治理、华阳河西隔堤整险加固，以及武汉新港江北铁路和快速通道黄冈段、随(州)麻(城)安(庆)铁路、武穴和蕲春长江大桥、团风麻城黄梅和罗田抽水蓄能电站等项目前期工作。

　　提高招商引资质效。完善招商引资工作机制，进一步规范项目洽谈、项目准入、规划选址、深度评审、综合审批、组织签约、项目建设、项目督办等重点环节，着力引进一批支撑作用强、技术含量高、投资强度大、产出效益好的项目。强化签约项目跟踪服务，完善督办考核机制，建立项目建设“绿色通道”，促进项目早开工、快建设、速见效。

　　大力推动全民创业。建立发展民营经济考核机制和激励机制，放宽准入门槛，鼓励引导非公有资本进入金融服务、公用事业、基础设施等国家没有明文禁止的一切领域，并做到一视同仁。进一步完善行政服务网络，积极打造公平、公正、公开、可预期、高效率的投资创业环境。大力实施回归工程，引导务工人员回乡创业。力争今年新增私营企业2000户以上、个体工商户2万户以上。

　　认真编制“十二五”规划。深入研究事关黄冈长远发展的全局性、战略性、关键性问题，集思广益、群策群力，切实做好规划编制工作，增强规划的前瞻性、针对性和可操作性。

　　提升新型工业化水平。推进产业规模化。坚持工业强市战略，继续实施“六个一百”工程，组织实施80个新增产值5000万元以上的项目，重点抓好伊利二期、李时珍药业二期、亚东水泥、红安雨润等33个过亿元项目。继续支持广济药业、祥云化工、华新水泥、汇源果汁、鸿路钢构、开阳星造船、大别山电厂、红安娃哈哈等骨干企业做大做强。开展“市场主体增量行动”，力争新增规模以上企业150家、销售收入过亿元企业20家、税收过千万元企业10家。

　　推进产业集群化。以重点企业为依托，集中力量培育配套紧密度高、集聚效应明显、市场竞争力强、产业延伸链条长的产业体系，在抓好食品饮料、纺织服装、医药化工、汽车配件、建筑建材等传统产业的同时，加快培育造船、窑炉、陶瓷、钢构、森工等新兴产业集群。

　　推进产业园区化。重点抓好工业园区和乡镇工业集中区建设，加快完善基础设施建设，理顺园区管理体制，在开发上实行“整合、优化、扩容”三同步，在布局上实现“集聚、集群、集约”三提高，在功能上实现“政务、商务、服务”三配套，着力打造发展速度快、质量效益好、税收贡献大、充满生机与活力的经济发展板块。

　　推进产业高新化。实施科技投融资、产学研结合、创新型企业建设、农业科技创新体系建设、科技型中小企业成长路线图五大专项，进一步推动市校合作，加快科研成果与专利技术产业化步伐，鼓励企业淘汰落后的设备、工艺和技术，提高产出效益。深入实施“质量兴市”战略，切实提高产品质量、工程质量、服务质量和环境质量，抓好品牌质量建设和优质产品生产基地建设，培育一批名牌产品和拥有知名品牌的大型企业，继续争创中国名牌和驰名商标。

　　突破性发展现代服务业。充分发挥现代金融对经济发展的支撑作用，落实更有针对性的政策措施，吸引金融机构落户黄冈，创新更多金融产品。加大信贷平台、融资平台、担保平台建设力度，进一步深化政银企合作，鼓励大型银行扩大信贷投入，抓紧组建新型农村金融机构，支持邮储银行发展资产业务，切实缓解融资难题。鼓励支持商业保险加快发展。举全市之力办好首届中国·黄冈大别山旅游节，加快培植黄冈大别山旅游开发公司，全力推进大别山旅游公路建设，加大地热资源开发力度，加强旅游资源整合、景点景区建设和旅游招商促销工作，深入开展湖北旅游名镇、名村和旅游强县(市)创建活动，推动旅游业快速发展。加快利用信息技术改造提升传统商贸流通业，大力发展现代物流业。实施“万村千乡市场工程”、“双百市场工程”和“新农村现代流通服务网络工程”，加强“农超对接”，落实家电、农机、汽车和摩托车下乡政策。加大信息资源整合力度，促进电信网、互联网和广电网“三网融合”。整顿规范房地产市场秩序，引导房地产业稳健发展，支持居民住房消费。

　　夯实“三农”发展基础。全面提高农业综合生产能力。在稳定粮食播种面积的基础上，大规模推进土地整理、复垦开发和低产田改造，大规模开展粮棉油高产创建活动，大规模实施畜牧水产和园艺作物标准化生产，继续推进农业板块基地和专业大县(市、区)建设，加快发展茶叶、板栗、药材、奶牛、森工等特色产业。力争千头奶牛牧场总数达到35个、存栏3万头，小龙虾养殖面积发展到40万亩，植树造林30万亩，完成林浆纸原料林建设任务;农村专业合作组织发展到1000家;新增绿色无公害农产品认证30个，创建省级以上农产品品牌10个。加快推进农业产业化经营。重点扶持3个国家级农产品加工示范园及32家省级以上重点龙头企业，力争建成年产值过30亿元的加工大县(市、区)2个，年产值过15亿元的加工园区1个，年销售收入过10亿元的加工型龙头企业3家。扎实推进新农村建设。认真落实国家支农惠农政策，加大农村饮水安全、道路、危房改造、沼气利用等涉及民生的农村基础设施建设投入。继续加强水资源管理和水利基础设施建设，大力推进大中型灌区续建配套和节水改造、排涝泵站更新改造和小型农田水利建设。进一步加大河道采砂管理力度。启动新一轮农村电网改造，抓好“一建三改”和绿色家园工程建设。大力推进农业标准化和农业机械化。进一步加强黄冈市区“菜篮子工程”建设。加大扶贫开发力度，深入开展“1321工程”、“双强工程”、“结对帮扶工程”和村企共建工程，对427个贫困村实施整村推进，解决8万贫困人口的脱贫问题。切实抓好红安县全国革命老区建设示范试点、麻城市中央彩票公益金项目试点、罗田县全国“两项制度”衔接试点、英山县全省山区脱贫奔小康试点以及全省新农村建设乡镇试点工作。

　　四、深入推进综合配套改革试验，增强发展动力与活力

　　高水平打造“两型社会”先行区。进一步完善临港经济区规划，积极争取和抓紧落实重点建设项目，加快黄冈高新区以及有关产业园区发展。高标准抓好麻城经济开发区武汉城市圈园区互动发展试点、罗田“两型社会”建设旅游体制综合配套改革试点、黄州“两型社会”建设再生资源综合利用示范区、团风“两型社会”建设承接产业转移示范区、田镇“两型社会”建设循环经济试验区、总路咀“两型社会”建设综合配套改革试验示范镇等试点工作，支持浠水、黄石跨江联合开发，力争在承接产业转移、壮大产业集群、发展循环经济、推进节能减排、统筹城乡发展、创新体制机制等方面有新突破，使其真正成为“两型社会”建设综改试验区的示范样板。

　　深化各项改革。围绕转变职能、理顺关系、优化结构、提高效能，全面完成新一轮市县政府机构改革任务。加强国有资产监管，切实履行出资人职责，扎实推进市直机关企业和经济实体新一轮脱钩改制工作。继续抓好企业上市工作。进一步稳定完善农村土地承包经营制度，建设土地流转市场，推进土地有序流转。继续抓好城中村、城郊村、园中村集体资产产权制度改革。积极推进集体林权制度改革，确保集体林权确权到户。落实最严格的耕地保护和节约集约用地制度，优化土地利用结构和布局。合理利用和有序开采矿产资源，推进资源性产品价格和环保收费改革，开展排污权交易、排污许可证制度试点工作。深化市厅合作机制，落实共建任务，放大共建效应，最大限度地聚合各种发展资源。深化财税体制改革，优化财政支出结构，加大对民生领域和社会事业、重点改革、重点项目建设和产业结构调整的支持保障力度。依法加强税收征管，继续落实结构性减税政策，确保全面完成年度收入目标。

　　大力实施开放先导战略。坚持对内对外开放并举，扩大出口比重与优化出口结构并重、引进资金技术与引进人才并行、引进来与走出去相结合，全面做好开放工作。转变外贸发展方式，增加自有品牌、自主技术及标准出口比重，突出抓好重点产业、重点企业、重点产品的出口工作，努力促进出口稳步回升。大力实施“走出去”战略，积极推进境外投资、劳务合作和工程承包。

　　继续推进节能减排。严格执行节能环保目标责任制，强化排污许可证管理，加大重点排污企业污染减排力度，突出抓好城镇污水处理厂的建设和运行管理、燃煤电厂脱硫设施的运行管理，确保各县市区污水处理厂建成并运行。继续实施十大节能工程，积极推进工业、交通、建筑三大领域节能，加快淘汰建材、冶金等行业的落后生产能力。深入开展省级环保模范城市、生态县市、环境优美乡镇、生态村以及生态文明单位创建活动。重点对全市主要河流、湖泊、水库进行综合整治及生态修复，确保“碧水行动”三年计划顺利实施。加大长河、遗爱湖环境综合治理力度，切实改善区域生态环境。完成经济开发区(工业园)区域环评工作。大力推行环保“绿色审批”制度，严把环境准入关。抓好生态公益林保护和退耕还林工作。抓好大别山(黄冈)国家地质公园和龙感湖国家级湿地保护区建设。加强农村环境综合整治，防治农业面源污染，提高“清洁家园”建设质效。

　　五、坚持高昂市区龙头，统筹区域经济发展

　　拓宽市区发展空间。立足当前，着眼长远，把优化区域发展布局、加快市区城市建设、提升城市带动功能，作为突破资源、空间制约的战略举措，作为调结构、扩内需、保增长，加快发展方式转变的重要抓手，作为推动工业化与城市化互动并进、经济社会又好又快发展的强力引擎。加快建设各具功能特色的产业园区和生态环境优美的商居区，全力打造“一河(三台河)两湖(遗爱湖，白潭湖)、滨江显山、山水园林、生态宜居”的新城区。坚持高起点规划、高标准建设、大力度推进，抓紧编制完善新城区规划尤其是三台河以东、城际铁路沿线及3个站区的规划，抓紧推进旧城改造和新旧城区对接通道等基础设施建设，抓紧做好土地收储和城市经营管理，加大“四城联创”工作力度，巩固省级卫生城市创建成果，推进中国优秀旅游城市目的地建设，有序开展省级文明城市和省级园林城市创建工作，进一步提高城市建设管理水平。

　　全面推进城镇化建设。进一步理顺城镇建设管理体制机制，完善城乡建设考核体系，大胆创新投融资方式，形成合力推进城镇化的工作格局。突出重点加强县市城区建设，加大基础设施建设投入，切实提升县市城区形象。抓紧编制城镇化和村庄建设规划，加强道路、绿化、污水处理和垃圾处理等基础设施建设，加大中心城镇、重点镇、口子镇、旅游名镇开发力度。重点建设20个生态文明镇、200个生态文明村。

　　推进区域经济竞相发展。完善市区一体化体制机制，增强市区发展活力，确保市区主要经济指标增幅持续高于全市平均水平。加大南湖工业园、黄冈化工园和禹王工业园建设力度，加快黄冈高新区产业孵化器建设，抓好华海造船、南华造船二期、北环电子、康友医疗器械、彩虹LED、青江化工、中粮集团油料加工等重点项目。加快发展县域经济，突出抓好工业园区和重点项目建设，培育一批规模以上企业和精品名牌;加强现代农业与纺织、食品等行业的产业链衔接，努力形成一批有市场竞争力的农产品加工龙头企业;着力抓好沿江船舶工业区，打造中部特种船舶制造基地和修配基地。坚持特色化、差异化发展策略，在铁路、公路沿线建设一批示范基地、专业市场、龙头加工企业和新型集镇。

　　六、大力发展社会事业，增强城市软实力

　　优先发展教育事业。优化教育资源布局规划与调整，健全教育投入保障机制和教师绩效考核机制，加快中等职业学校整合建设步伐，加强薄弱学校改造，抓紧实施校舍安全工程。加强师德建设，提高教师专业水平。重视民办教育，发展学前教育、特殊教育、远程教育，支持高校加快发展。

　　提高医疗卫生服务水平。积极实施国家基本药物制度，着力减轻群众基本药物费用负担。加强公共卫生服务体系建设，提高应对突发公共卫生事件的能力。继续加强血吸虫病和甲型H1N1流感、手足口病、艾滋病、结核病等疫病防控工作。进一步完善农村新型合作医疗制度，健全基层医疗卫生服务体系，促进基本公共卫生服务均等化。

　　增创文化发展优势。发掘红色文化、名人文化、佛教禅宗文化、黄梅戏剧文化等特色资源，打造一批精品力作。支持促进娱乐演出、影视制作、电影放映、音像制品、艺术教育、印刷发行等产业做大做强。加大文化产业改革力度，培植新的文化市场主体，建设一批有竞争力的文化产业企业。深入贯彻《全民健身条例》，积极备战第13届省运会，进一步加快体育事业发展。

　　加强民主法制和精神文明建设。扎实抓好文明村镇、文明社区、文明单位评创工作，不断提高市民文明素质和社会文明程度。加强法制宣传和法律援助工作，深入推进“法治黄冈”建设，全面落实“五五”普法任务。坚持计划生育基本国策，综合整治出生人口性别比，着力提高人口素质。全面实施妇女儿童发展规划，确保终期评估达标。加强城乡社区建设，完善社区公共服务设施。加强国防动员，深化国防教育，支持驻黄解放军和武警部队建设，不断提高预备役部队、民兵和人民防空建设水平。积极开展双拥共建活动，巩固军政军民团结。

　　七、切实保障和改善民生，提高人民群众生活水平

　　努力扩大就业。实施更加积极的就业政策，引导和促进劳动密集型企业、中小企业、民营企业和各种服务业加快发展，创造更多的就业岗位。认真落实扶持就业政策，加强职业技能培训，开展“一帮一”创业活动，鼓励和支持劳动者自主创业，以创业带动就业。加快建立服务、培训、维权三位一体的农村劳动力输出工作机制，加大回归创业引导力度。完善就业援助长效机制，健全公共就业服务体系，重点帮扶“零就业家庭”、“4050”、低保人员等困难群体实现再就业。

　　健全社会保障体系。坚持“保基本、广覆盖、多层次、可持续”，进一步扩大社会保险覆盖面，提高保障水平，重点促进农民工、非公有制经济组织就业人员、个体工商户、灵活就业人员参保。继续加强社会保险费征缴和监管。认真做好城镇企业职工养老保险省级统筹，抓好城镇企业职工基本养老保险关系转移接续工作。加大新农保试点工作力度，落实被征地农民社会保障政策措施。做好企业退休人员基本养老金调标工作。维护企业职工和农民工的合法权益，促进劳资关系和谐稳定。健全城乡低保动态管理机制，逐步提高保障标准，适度扩大贫困地区低保覆盖面。建立健全困难群众临时救助和医疗救助制度，完善城乡社会救助体系。认真落实农村“五保”、孤儿供养政策。强化优抚安置工作，加快慈善事业发展。加大住房公积金归集、使用和监管力度，确保资金保值增值。切实加强库区移民后扶项目建设和管理，确保完成南水北调移民安置任务。

　　维护社会和谐稳定。严格落实稳定工作“第一责任”，完善领导干部处理群众来信来访和接待信访群众制度，建立大调解工作格局，健全矛盾预警机制、应急处置机制、风险评估机制和矛盾纠纷调处机制，主动防范和妥善化解社会矛盾。加强国家安全工作。加强社会治安综合治理，大力推进社会治安视频监控体系建设，积极预防青少年犯罪，依法打击各种刑事犯罪活动，不断增强人民群众的安全感。加强应急预案体系、应急队伍和应急平台建设，提高应急管理水平。加强预防预警，切实抓好防洪、抗震、防治地质灾害、应对极端气候变化等防灾减灾工作。严格落实安全生产责任制，加大安全生产执法、治理和宣传教育力度，有效防范和坚决遏制重特大安全事故。高度重视消防工作，增强全社会消防安全意识。强化农产品、食品药品和特种设备质量安全监管，依法加强市场物价管理。严格统计和调查监测评价考核，提高数据质量。做好第六次人口普查。继续做好二轮志书编修和年鉴编撰工作。以创建“和谐寺观教堂”和做好城市流动少数民族人口工作为重点，加强民族宗教工作。认真做好外事、侨务、对台、保密等工作，重视支持老龄和残疾人事业。

　　努力为人民群众办实事。进一步加大投入、强化责任、落实措施，着力解决好人民群众普遍关心的实际问题：(1)城镇新增就业4万人，培训劳动力30万人次。(2)新增廉租住房15万平方米。(3)新建通村水泥(沥青)公路1500公里。(4)再解决农村40万人饮水安全问题。(5)新增农村低保对象5万人;启动建设黄冈市精神病医院综合楼。(6)推进清洁能源入户工程，新建农村户用沼气6.5万户。(7)实施100个村的“农民体育健身工程”;再建标准农家书屋600家、乡镇综合文化站8个;新建标准农家店500个、供销社村级综合服务社600个。(8)再建成农村合格学校153所。(9)为500名0—6岁贫困残疾儿童实施抢救性康复救助;为困难群众和农民工办理法律援助案件1000件、法律援助事项10000件以上。(10)全面启动和加快建设黄冈市体育中心、黄梅戏大剧院、博物馆、老年大学和中等职业技术学校。

　　八、着力提高行政能力，努力建设人民满意政府

　　面对经济社会发展繁重任务，政府要切实改进工作，加强自身建设，努力打造公信力高、执行力强、工作作风硬、群众信得过的人民满意政府。

　　转变政府职能。优化政府组织结构，加快推进政企、政资、政事和政府与社会中介组织分开。探索实施政府公共服务外包制，推进事业单位分类改革，更加注重全面履行职能，把该管的切实管好、管到位，把不该管的真正放下去，推动政府职能进一步向创造良好发展环境、提供优质公共服务、维护社会公平正义转变，最大限度地激发社会发展活力。

　　提高发展能力。将解放思想与解决问题结合起来，下大力突破观念瓶颈、资金瓶颈、能力瓶颈，使思想观念始终与科学发展观的要求相适应，不断提高战略思维、创新思维、辩证思维能力，提高谋划发展、统筹发展、优化发展、推动发展的本领，增强群众工作、公共服务、社会管理、维护稳定的本领。

　　严格依法行政。主动接受人大及其常委会的法律监督、工作监督和政协的民主监督，重视社会监督和舆论监督，坚持依法行政，建设法治政府。认真办理人大代表议案、建议、批评、意见和政协委员提案，创新工作方法，提高承办质量。加强电子政务建设，推行网上办事，扩大政务公开，提高政府透明度。严格落实行政问责制，对已明确的任务、确定的事项，既定责到人，又问责到位，做到有权必有责、有责必问责、有错必究责。进一步压缩和优化审批流程，提高办事效率和服务质量。建设诚信政府，慎诺重践，言出必行，以提高政府公信力推动诚信社会建设。

　　坚持勤政廉政。进一步健全政府系统惩治和预防腐败体系建设，严格执行党风廉政建设责任制，健全权力运行制约和监督机制，努力营造干净干事、公正干事、廉洁干事的政务环境。进一步加大对重点领域、重点部门、重点项目、重点资金的审计监督力度。始终保持艰苦奋斗的光荣传统，从严控制车辆、会议、公务接待、出国等公务消费，努力降低行政成本。进一步优化经济发展环境，坚决纠正损害群众利益的不正之风，着力解决行政不作为、乱作为、效能低下等问题，为经济社会发展创造良好环境。

　　各位代表!做好今年的工作，任务繁重艰巨，责任重大光荣。让我们更加紧密地团结在以胡锦涛同志为总书记的党中央周围，在市委正确领导下，弘扬老区精神，满怀发展激情，焕发昂扬斗志，同心同德，奋发向上，为全面完成今年各项任务、推动黄冈科学发展新跨越而努力奋斗!
